# (1*E*,4*E*)-1,5-Bis[4-(di­ethyl­amino)­phen­yl]penta-1,4-dien-3-one

**DOI:** 10.1107/S1600536814008356

**Published:** 2014-04-26

**Authors:** Pumsak Ruanwas, Suchada Chantrapromma, Hazem A. Ghabbour, Hoong-Kun Fun

**Affiliations:** a Department of Chemistry and Center of Excellence for Innovation in Chemistry, Faculty of Science, Prince of Songkla University, Hat-Yai, Songkhla 90112, Thailand; b Department of Chemistry, Faculty of Science, Prince of Songkla University, Hat-Yai, Songkhla 90112, Thailand; c Department of Pharmaceutical Chemistry, College of Pharmacy, King Saud University, Riyadh 11451, Kingdom of Saudi Arabia; d X-ray Crystallography Unit, School of Physics, Universiti Sains Malaysia, 11800 USM, Penang, Malaysia

## Abstract

There are two crystallograpically independent mol­ecules in the asymmetric unit of the title bis­chalcone derivative, C_25_H_32_N_2_O. Both mol­ecules are twisted with a dihedral angle between the two substituted benzene rings of 11.19 (16)° in one mol­ecule and 14.40 (15)° in the other. The central penta-1,4-dien-3-one fragments make dihedral angles of 8.49 (17) and 4.26 (17)° with the two adjacent benzene rings in one mol­ecule, whereas the corresponding values are 8.42 (16) and 6.18 (16)° in the other. In the crystal, mol­ecules are arranged into chains along the *c-*axis direction. Adjacent chains are inter-linked by weak inter­molecular C—H⋯O inter­actions. The crystal is further stabilized by C—H⋯π inter­actions.

## Related literature   

For bond-length data, see: Allen *et al.* (1987 ▶). For related structures, see: Fun *et al.* (2010 ▶); Harrison *et al.* (2006 ▶); Ruanwas *et al.* (2011 ▶). For background to and applications of bis­chalcones, see: Barnabas *et al.* (1992 ▶); Makarov *et al.* (2012 ▶); Shibata *et al.* (2009 ▶); Wanare *et al.* (2010 ▶); Weber *et al.* (2005 ▶); Zhao *et al.* (2010 ▶)
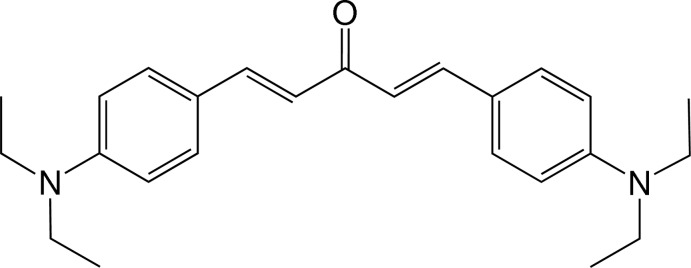


## Experimental   

### 

#### Crystal data  

C_25_H_32_N_2_O
*M* *_r_* = 376.53
Monoclinic, 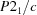

*a* = 10.4290 (4) Å
*b* = 40.4773 (16) Å
*c* = 10.8087 (5) Å
β = 100.2621 (13)°
*V* = 4489.8 (3) Å^3^
*Z* = 8
Mo *K*α radiation
μ = 0.07 mm^−1^
*T* = 296 K
0.42 × 0.38 × 0.34 mm

#### Data collection  

Bruker APEXII D8 Venture diffractometer
Absorption correction: multi-scan (*SADABS*; Bruker, 2009 ▶) *T*_min_ = 0.972, *T*_max_ = 0.977
79317 measured reflections
10289 independent reflections
5710 reflections with *I* > 2σ(*I*)
*R*_int_ = 0.037

#### Refinement  

*R*[*F* ^2^ > 2σ(*F* ^2^)] = 0.083
*wR*(*F* ^2^) = 0.270
*S* = 1.03
10289 reflections
501 parameters
H-atom parameters constrained
Δρ_max_ = 0.73 e Å^−3^
Δρ_min_ = −0.50 e Å^−3^

### 

Data collection: *APEX2* (Bruker, 2009 ▶); cell refinement: *SAINT* (Bruker, 2009 ▶); data reduction: *SAINT*; program(s) used to solve structure: *SHELXTL* (Sheldrick, 2008 ▶); program(s) used to refine structure: *SHELXTL*; molecular graphics: *Mercury* (Macrae *et al.*, 2006 ▶); software used to prepare material for publication: *SHELXTL*, *PLATON* (Spek, 2009 ▶), *Mercury* (Macrae *et al.*, 2006 ▶) and *publCIF* (Westrip, 2010 ▶).

## Supplementary Material

Crystal structure: contains datablock(s) global, I. DOI: 10.1107/S1600536814008356/sj5396sup1.cif

Structure factors: contains datablock(s) I. DOI: 10.1107/S1600536814008356/sj5396Isup2.hkl

Supporting information file. DOI: 10.1107/S1600536814008356/sj5396Isup3.cml

CCDC reference: 997066

Additional supporting information:  crystallographic information; 3D view; checkCIF report

## Figures and Tables

**Table 1:** Hydrogen-bond geometry (Å, °) *Cg*3 and *Cg*4 are the centroids of the C1*B*–C6*B* and C12*B*–C17*B* rings, respectively.

*D*—H⋯*A*	*D*—H	H⋯*A*	*D*⋯*A*	*D*—H⋯*A*
C8*B*—H8*BA*⋯O1*B* ^i^	0.93	2.59	3.479 (4)	160
C16*A*—H16*A*⋯*Cg*3^ii^	0.93	2.91	3.758 (4)	152
C21*A*—H21*A*⋯*Cg*4^iii^	0.96	2.79	3.541 (5)	136

Symmetry codes: (i) 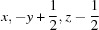; (ii) 
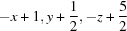
; (iii) 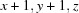.

**Fig. 1.**
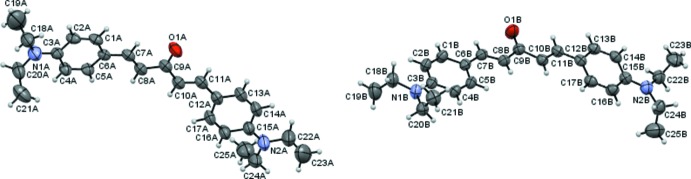
The molecular structure of the title compound, drawn with 30% probability displacement ellipsoids and the atom-numbering scheme.

**Fig. 2.**
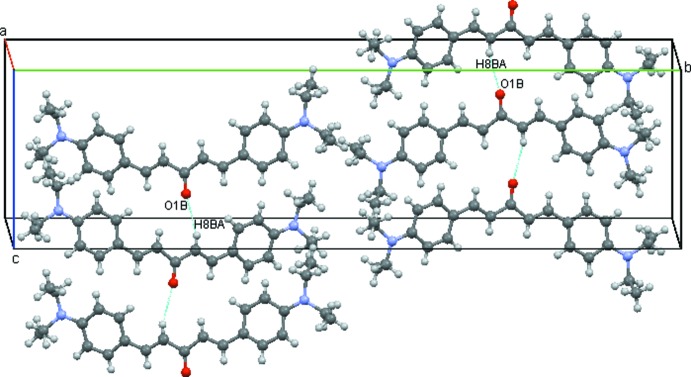
Intermolecular hydrogen bonding interactions in the title compound viewd appoximately along the a axis, showing chains along the c axis. Hydrogen bonds are shown as dashed lines.

## supplementary crystallographic information

### 1. Comment

Mono-carbonyl analogues of curcumin are an important class of compounds due to their variety of properties. For example these compounds exhibit anti-inflammatory (Zhao *et al.*, 2010), antimalarial (Wanare *et al.*, 2010), antitumor (Shibata *et al.*, 2009) and anti-oxidant properties (Weber *et al.*, 2005). They also act as dye sensitizers (Barnabas *et al.*, 1992) and fluorescence agents (Makarov *et al.*, 2012). These analogues were designed to counteract some of the disadvantageous properties of curcumin such as its poor bioavailability and instability in neutral to basic conditions. We have previously reported the crystal structures of (1*E*,4*E*)-1,5-bis(2,4,5-trimethoxyphenyl)penta-1,4-dien-3-one (I) (Fun *et al.*, 2010) and (1*E*,4*E*)-1,5-bis(2,4,6-trimethoxyphenyl)penta-1,4-dien-3-one (II) (Ruanwas *et al.*, 2011). The title compound (III) is one of the mono-carbonyl analogues of curcumin designed and synthesized by our group to study anti-tyrosinase activity and its fluorescence properties. It was found that the title compound exhibits fluorescence properties with an orange fluorescence color which will be reported elsewhere with its closely related compounds, it also possesses anti-tyrosinase activity by the dopachrome method with an IC_50_ value of 0.018 mg ml^-1^. We reported herein the crystal structure of (III).

There are two crystallographically independent molecules *A* and *B* in the asymmetric unit of (III) (Fig. 1) with the same conformation but slight differences in bond angles. The molecular structure of (III), C_25_H_32_N_2_O is unsymmetrical and twisted. The dihedral angle between the C1–C6 and C12–C17 benzene rings is 11.19 (16)° in molecule *A* (Fig. 2a) whereas it is 14.40 (15)° in molecule *B*. The central penta-1,4-dien-3-one unit (C7–C11/O1) is planar with *r.m.s.* deviations 0.0463 (3) and 0.0357 (3) Å for molecules *A* and *B*, respectively. The mean plane through this central unit makes dihedral angles of 8.49 (17) and 4.26 (17)° with the two adjacent C1–C6 and C12–C17 benzene rings, respectively in molecule *A* whereas the corresponding values are 8.42 (16) and 6.18 (16)° in molecule *B*. The two ethyl groups of each diethylamino substituent in both molecules *A* and *B* deviate from the molecular plane and point to opposite sides of the molecule to reduce the steric hindrance between them with the torsion angles C3–N1–C18–C19 = 104.1 (5)°, C3–N1–C20–C21 = 79.5 (5)°, C15–N2–C22–C23 = 106.8 (6)° and C15–N2–C24–C25 = 75.1 (6)° in molecule *A*. The corresponding values are 96.1 (4), 79.9 (4), 90.3 (4) and 97.6 (5)° in molecule *B*. The bond distances are in normal ranges (Allen *et al.*, 1987) and are comparable with those found in related structures (Fun *et al.*, 2010; Harrison *et al.*, 2006 and Ruanwas *et al.*, 2011).

In the crystal packing (Fig. 2), the molecules are arranged into chains along the *c* axis and the adjacent chains are further linked by weak C—H···O interactions (Table 1). The crystal is further stabilized by weak intermolecular C—H···π interactions (Table 1). Interestingly there are only one C—H···O and two C—H···π interactions stabilising the structure of (III). This contrasts sharply with the packing for (I) and (II) where significantly more weak C—H···O and C—H···π interactions were observed (Fun *et al.*, 2010 and Ruanwas *et al.*, 2011).

### 2. Experimental

The title compound was synthesized by mixing 4-diethylaminobenzaldehyde (0.90 g, 6 mmol) and acetone (0.25 ml, 3 mmol) in ethanol (30 ml). 30% NaOH aqueous solution (5 ml) was then added and the mixture was stirred at room temperature for 2 h, The resulting orange solid obtained was collected by filtration, washed with distilled water and dried. Orange block-shaped single crystals of the title compound were grown in ethanol by slow evaporation, Mp. 440–441 K.

### 3. Refinement

All H atoms were positioned geometrically and allowed to ride on their parent atoms, with d(C—H) = 0.93 Å for aromatic and CH; 0.96 Å for CH_3_ atoms. The *U*_iso_ values were constrained to be 1.5*U*_eq_ of the carrier atom for methyl H atoms and 1.2*U*_eq_ for the remaining H atoms. A rotating group model was used for the methyl groups. The same *U_ij_* parameters were used for atom pairs N1A/C18A and N2B/C22B.

### Crystal data

**Table:**

C_25_H_32_N_2_O	*D*_x_ = 1.114 Mg m^−^^3^
*M**_r_* = 376.53	Melting point = 440–441 K
Monoclinic, *P*2_1_/*c*	Mo *K*α radiation, λ = 0.71073 Å
*a* = 10.4290 (4) Å	Cell parameters from 10289 reflections
*b* = 40.4773 (16) Å	θ = 2.2–27.5°
*c* = 10.8087 (5) Å	µ = 0.07 mm^−^^1^
β = 100.2621 (13)°	*T* = 296 K
*V* = 4489.8 (3) Å^3^	Block, orange
*Z* = 8	0.42 × 0.38 × 0.34 mm
*F*(000) = 1632	

### Data collection

**Table:**

Bruker APEXII D8 Venture diffractometer	5710 reflections with *I* > 2σ(*I*)
φ and ω scans	*R*_int_ = 0.037
Absorption correction: multi-scan (*SADABS*; Bruker, 2009)	θ_max_ = 27.5°, θ_min_ = 2.2°
*T*_min_ = 0.972, *T*_max_ = 0.977	*h* = −13→13
79317 measured reflections	*k* = −52→52
10289 independent reflections	*l* = −14→10

### Refinement

**Table:**

Refinement on *F*^2^	Primary atom site location: structure-invariant direct methods
Least-squares matrix: full	Secondary atom site location: difference Fourier map
*R*[*F*^2^ > 2σ(*F*^2^)] = 0.083	Hydrogen site location: inferred from neighbouring sites
*wR*(*F*^2^) = 0.270	H-atom parameters constrained
*S* = 1.03	*w* = 1/[σ^2^(*F*_o_^2^) + (0.107*P*)^2^ + 2.8109*P*] where *P* = (*F*_o_^2^ + 2*F*_c_^2^)/3
10289 reflections	(Δ/σ)_max_ = 0.001
501 parameters	Δρ_max_ = 0.73 e Å^−^^3^
0 restraints	Δρ_min_ = −0.50 e Å^−^^3^

### Special details

**Table:**

Geometry. All e.s.d.'s (except the e.s.d. in the dihedral angle between two l.s. planes) are estimated using the full covariance matrix. The cell e.s.d.'s are taken into account individually in the estimation of e.s.d.'s in distances, angles and torsion angles; correlations between e.s.d.'s in cell parameters are only used when they are defined by crystal symmetry. An approximate (isotropic) treatment of cell e.s.d.'s is used for estimating e.s.d.'s involving l.s. planes.

### Fractional atomic coordinates and isotropic or equivalent isotropic displacement parameters (Å^2^)

**Table:**

	*x*	*y*	*z*	*U*_iso_*/*U*_eq_	
O1A	0.1268 (2)	0.96078 (6)	0.7957 (3)	0.1055 (9)	
N1A	0.4981 (3)	1.14078 (9)	0.9984 (4)	0.1106 (9)	
N2A	0.3434 (3)	0.78543 (9)	0.4536 (4)	0.1131 (12)	
C1A	0.2678 (3)	1.07418 (8)	0.9275 (3)	0.0696 (8)	
H1AA	0.1786	1.0713	0.9230	0.084*	
C2A	0.3180 (3)	1.10444 (8)	0.9626 (3)	0.0713 (8)	
H2AA	0.2637	1.1211	0.9826	0.086*	
C3A	0.4464 (3)	1.11050 (7)	0.9686 (3)	0.0664 (7)	
C4A	0.5193 (3)	1.08345 (8)	0.9393 (3)	0.0717 (8)	
H4AA	0.6082	1.0863	0.9418	0.086*	
C5A	0.4671 (3)	1.05332 (8)	0.9075 (3)	0.0661 (8)	
H5AA	0.5215	1.0362	0.8917	0.079*	
C6A	0.3383 (3)	1.04736 (7)	0.8981 (3)	0.0584 (7)	
C7A	0.2772 (3)	1.01645 (8)	0.8583 (3)	0.0656 (8)	
H7AA	0.1883	1.0161	0.8597	0.079*	
C8A	0.3216 (3)	0.98812 (8)	0.8197 (3)	0.0679 (8)	
H8AA	0.4098	0.9865	0.8159	0.081*	
C9A	0.2389 (3)	0.95964 (8)	0.7835 (3)	0.0716 (8)	
C10A	0.2904 (3)	0.93036 (8)	0.7311 (3)	0.0704 (8)	
H10A	0.3791	0.9298	0.7289	0.085*	
C11A	0.2197 (3)	0.90447 (8)	0.6866 (3)	0.0705 (8)	
H11A	0.1328	0.9057	0.6958	0.085*	
C12A	0.2524 (3)	0.87467 (8)	0.6263 (3)	0.0670 (8)	
C13A	0.1631 (3)	0.85018 (9)	0.5856 (3)	0.0788 (9)	
H13A	0.0781	0.8533	0.5983	0.095*	
C14A	0.1903 (3)	0.82147 (9)	0.5276 (3)	0.0830 (10)	
H14A	0.1235	0.8064	0.5019	0.100*	
C15A	0.3124 (3)	0.81444 (9)	0.5066 (3)	0.0816 (9)	
C16A	0.4024 (3)	0.83956 (9)	0.5449 (4)	0.0874 (10)	
H16A	0.4871	0.8368	0.5307	0.105*	
C17A	0.3733 (3)	0.86792 (8)	0.6019 (3)	0.0780 (9)	
H17A	0.4391	0.8834	0.6252	0.094*	
C18A	0.4105 (4)	1.17228 (11)	0.9994 (5)	0.1106 (9)	
H18A	0.4459	1.1910	0.9608	0.133*	
H18B	0.3220	1.1682	0.9565	0.133*	
C19A	0.4157 (7)	1.1772 (2)	1.1270 (6)	0.191 (3)	
H19A	0.3680	1.1969	1.1397	0.287*	
H19B	0.5048	1.1797	1.1676	0.287*	
H19C	0.3778	1.1586	1.1620	0.287*	
C20A	0.6287 (4)	1.14794 (10)	0.9907 (5)	0.1053 (14)	
H20A	0.6831	1.1294	1.0234	0.126*	
H20B	0.6571	1.1670	1.0427	0.126*	
C21A	0.6470 (4)	1.15506 (13)	0.8543 (6)	0.1359 (19)	
H21A	0.7367	1.1602	0.8536	0.204*	
H21B	0.5934	1.1735	0.8216	0.204*	
H21C	0.6221	1.1359	0.8031	0.204*	
C22A	0.2518 (5)	0.75844 (13)	0.4196 (6)	0.1341 (19)	
H22A	0.2928	0.7379	0.4519	0.161*	
H22B	0.1766	0.7619	0.4594	0.161*	
C23A	0.2087 (6)	0.7554 (2)	0.2853 (6)	0.192 (3)	
H23A	0.1598	0.7353	0.2675	0.288*	
H23B	0.2830	0.7549	0.2442	0.288*	
H23C	0.1545	0.7739	0.2551	0.288*	
C24A	0.4748 (4)	0.77697 (11)	0.4505 (5)	0.1150 (15)	
H24A	0.5193	0.7960	0.4232	0.138*	
H24B	0.4770	0.7593	0.3901	0.138*	
C25A	0.5461 (5)	0.76578 (13)	0.5796 (6)	0.1391 (19)	
H25A	0.6335	0.7594	0.5738	0.209*	
H25B	0.5012	0.7473	0.6076	0.209*	
H25C	0.5485	0.7836	0.6385	0.209*	
O1B	0.1127 (3)	0.24045 (6)	1.2120 (2)	0.0964 (8)	
N1B	0.3342 (3)	0.42416 (6)	0.9287 (2)	0.0746 (7)	
N2B	−0.0762 (3)	0.06584 (7)	0.7744 (3)	0.0836 (6)	
C1B	0.2823 (3)	0.35510 (8)	1.1411 (3)	0.0695 (8)	
H1BA	0.2950	0.3505	1.2267	0.083*	
C2B	0.3181 (3)	0.38536 (8)	1.1016 (3)	0.0696 (8)	
H2BA	0.3553	0.4008	1.1610	0.084*	
C3B	0.2996 (3)	0.39377 (7)	0.9702 (3)	0.0608 (7)	
C4B	0.2444 (3)	0.36941 (7)	0.8815 (3)	0.0641 (7)	
H4BA	0.2315	0.3740	0.7959	0.077*	
C5B	0.2105 (3)	0.33926 (7)	0.9227 (3)	0.0621 (7)	
H5BA	0.1756	0.3235	0.8636	0.074*	
C6B	0.2267 (3)	0.33099 (7)	1.0545 (3)	0.0585 (7)	
C7B	0.1895 (3)	0.29952 (7)	1.1018 (3)	0.0637 (7)	
H7BA	0.2029	0.2970	1.1887	0.076*	
C8B	0.1380 (3)	0.27394 (7)	1.0328 (3)	0.0628 (7)	
H8BA	0.1215	0.2756	0.9456	0.075*	
C9B	0.1068 (3)	0.24305 (8)	1.0936 (3)	0.0656 (7)	
C10B	0.0684 (3)	0.21543 (7)	1.0052 (3)	0.0642 (7)	
H10B	0.0672	0.2185	0.9197	0.077*	
C11B	0.0353 (3)	0.18616 (7)	1.0473 (3)	0.0635 (7)	
H11B	0.0329	0.1849	1.1327	0.076*	
C12B	0.0026 (3)	0.15601 (7)	0.9747 (3)	0.0584 (7)	
C13B	−0.0287 (3)	0.12771 (7)	1.0357 (3)	0.0627 (7)	
H13B	−0.0297	0.1286	1.1215	0.075*	
C14B	−0.0583 (3)	0.09850 (7)	0.9723 (3)	0.0631 (7)	
H14B	−0.0800	0.0802	1.0162	0.076*	
C15B	−0.0564 (3)	0.09543 (7)	0.8384 (3)	0.0653 (7)	
C16B	−0.0273 (3)	0.12416 (8)	0.7745 (3)	0.0712 (8)	
H16B	−0.0272	0.1235	0.6885	0.085*	
C17B	0.0009 (3)	0.15306 (8)	0.8405 (3)	0.0675 (8)	
H17B	0.0199	0.1717	0.7969	0.081*	
C18B	0.3718 (4)	0.45130 (9)	1.0227 (4)	0.0888 (10)	
H18C	0.3391	0.4722	0.9858	0.107*	
H18D	0.3316	0.4473	1.0956	0.107*	
C19B	0.5093 (5)	0.45352 (13)	1.0617 (5)	0.1251 (16)	
H19D	0.5293	0.4709	1.1226	0.188*	
H19E	0.5493	0.4583	0.9902	0.188*	
H19F	0.5420	0.4329	1.0985	0.188*	
C20B	0.3069 (3)	0.43348 (8)	0.7920 (3)	0.0752 (8)	
H20C	0.3648	0.4513	0.7779	0.090*	
H20D	0.3253	0.4147	0.7423	0.090*	
C21B	0.1729 (4)	0.44383 (10)	0.7499 (4)	0.0940 (11)	
H21D	0.1603	0.4495	0.6624	0.141*	
H21E	0.1546	0.4627	0.7977	0.141*	
H21F	0.1152	0.4261	0.7618	0.141*	
C22B	−0.0880 (4)	0.03430 (9)	0.8460 (3)	0.0836 (6)	
H22C	−0.0547	0.0159	0.8035	0.100*	
H22D	−0.0366	0.0360	0.9299	0.100*	
C23B	−0.2198 (4)	0.02852 (12)	0.8539 (5)	0.1128 (14)	
H23D	−0.2272	0.0078	0.8956	0.169*	
H23E	−0.2710	0.0278	0.7708	0.169*	
H23F	−0.2509	0.0460	0.9008	0.169*	
C24B	−0.0960 (4)	0.06305 (10)	0.6325 (3)	0.0905 (11)	
H24C	−0.1620	0.0464	0.6054	0.109*	
H24D	−0.1289	0.0840	0.5962	0.109*	
C25B	0.0196 (5)	0.05458 (15)	0.5839 (5)	0.1399 (19)	
H25D	−0.0022	0.0517	0.4945	0.210*	
H25E	0.0554	0.0344	0.6221	0.210*	
H25F	0.0826	0.0720	0.6028	0.210*	

### Atomic displacement parameters (Å^2^)

**Table:**

	*U*^11^	*U*^22^	*U*^33^	*U*^12^	*U*^13^	*U*^23^
O1A	0.0640 (15)	0.0930 (18)	0.167 (3)	−0.0138 (12)	0.0397 (16)	−0.0133 (17)
N1A	0.0909 (18)	0.0963 (19)	0.150 (3)	−0.0101 (14)	0.0372 (17)	−0.0256 (17)
N2A	0.085 (2)	0.093 (2)	0.164 (3)	−0.0271 (18)	0.030 (2)	−0.043 (2)
C1A	0.0505 (15)	0.079 (2)	0.083 (2)	0.0094 (14)	0.0213 (14)	0.0172 (17)
C2A	0.0662 (18)	0.068 (2)	0.086 (2)	0.0179 (15)	0.0298 (16)	0.0121 (16)
C3A	0.0608 (17)	0.0623 (18)	0.078 (2)	0.0055 (14)	0.0163 (14)	0.0012 (15)
C4A	0.0486 (15)	0.071 (2)	0.094 (2)	0.0055 (14)	0.0083 (15)	−0.0018 (17)
C5A	0.0465 (15)	0.0704 (19)	0.080 (2)	0.0135 (13)	0.0082 (13)	0.0019 (15)
C6A	0.0505 (15)	0.0649 (17)	0.0601 (16)	0.0065 (13)	0.0109 (12)	0.0124 (13)
C7A	0.0502 (15)	0.074 (2)	0.0728 (19)	0.0014 (14)	0.0128 (13)	0.0181 (15)
C8A	0.0539 (16)	0.071 (2)	0.080 (2)	−0.0022 (14)	0.0140 (14)	0.0118 (16)
C9A	0.0588 (18)	0.075 (2)	0.083 (2)	−0.0042 (15)	0.0173 (15)	0.0096 (16)
C10A	0.0582 (17)	0.073 (2)	0.080 (2)	−0.0022 (15)	0.0113 (15)	0.0097 (16)
C11A	0.0581 (17)	0.074 (2)	0.080 (2)	−0.0045 (15)	0.0130 (15)	0.0181 (17)
C12A	0.0573 (17)	0.0680 (19)	0.0731 (19)	−0.0080 (14)	0.0044 (14)	0.0130 (15)
C13A	0.0582 (18)	0.079 (2)	0.098 (2)	−0.0142 (16)	0.0114 (16)	0.0093 (19)
C14A	0.063 (2)	0.084 (2)	0.098 (3)	−0.0236 (17)	0.0041 (17)	0.001 (2)
C15A	0.071 (2)	0.078 (2)	0.095 (2)	−0.0172 (17)	0.0119 (17)	−0.0069 (19)
C16A	0.0618 (19)	0.083 (2)	0.118 (3)	−0.0147 (17)	0.0155 (18)	−0.010 (2)
C17A	0.0570 (18)	0.070 (2)	0.104 (3)	−0.0157 (15)	0.0064 (16)	0.0020 (18)
C18A	0.0909 (18)	0.0963 (19)	0.150 (3)	−0.0101 (14)	0.0372 (17)	−0.0256 (17)
C19A	0.185 (7)	0.266 (9)	0.112 (4)	−0.041 (6)	−0.001 (4)	0.006 (5)
C20A	0.074 (2)	0.077 (2)	0.162 (4)	−0.0083 (18)	0.015 (2)	−0.015 (3)
C21A	0.080 (3)	0.143 (4)	0.189 (6)	−0.003 (3)	0.037 (3)	0.031 (4)
C22A	0.112 (4)	0.131 (4)	0.164 (5)	−0.035 (3)	0.038 (3)	−0.054 (4)
C23A	0.145 (5)	0.288 (9)	0.141 (5)	−0.077 (6)	0.018 (4)	−0.055 (6)
C24A	0.100 (3)	0.099 (3)	0.157 (5)	−0.020 (2)	0.051 (3)	−0.039 (3)
C25A	0.120 (4)	0.123 (4)	0.179 (6)	0.011 (3)	0.041 (4)	−0.013 (4)
O1B	0.134 (2)	0.0927 (17)	0.0633 (14)	−0.0248 (15)	0.0186 (13)	−0.0008 (12)
N1B	0.0829 (18)	0.0649 (16)	0.0698 (16)	−0.0111 (13)	−0.0032 (13)	−0.0023 (13)
N2B	0.0940 (16)	0.0742 (14)	0.0833 (15)	−0.0112 (12)	0.0177 (12)	−0.0050 (11)
C1B	0.082 (2)	0.071 (2)	0.0533 (16)	−0.0018 (16)	0.0069 (14)	−0.0071 (14)
C2B	0.080 (2)	0.0655 (19)	0.0590 (17)	−0.0057 (15)	0.0018 (14)	−0.0119 (14)
C3B	0.0572 (16)	0.0609 (17)	0.0621 (17)	0.0046 (13)	0.0046 (13)	−0.0066 (13)
C4B	0.0693 (18)	0.0680 (19)	0.0514 (15)	0.0025 (14)	0.0006 (13)	−0.0048 (13)
C5B	0.0631 (17)	0.0613 (18)	0.0586 (17)	−0.0005 (13)	0.0021 (13)	−0.0118 (13)
C6B	0.0573 (15)	0.0621 (17)	0.0557 (16)	0.0044 (12)	0.0091 (12)	−0.0044 (13)
C7B	0.0647 (17)	0.0709 (19)	0.0559 (16)	0.0036 (14)	0.0119 (13)	−0.0056 (14)
C8B	0.0647 (17)	0.0656 (18)	0.0583 (16)	0.0015 (14)	0.0116 (13)	−0.0017 (14)
C9B	0.0678 (18)	0.0706 (19)	0.0590 (18)	−0.0025 (14)	0.0131 (14)	−0.0008 (14)
C10B	0.0646 (17)	0.0654 (18)	0.0631 (17)	−0.0018 (14)	0.0127 (13)	0.0044 (14)
C11B	0.0582 (16)	0.0703 (19)	0.0617 (17)	0.0000 (13)	0.0099 (13)	0.0069 (14)
C12B	0.0517 (15)	0.0583 (16)	0.0649 (17)	0.0045 (12)	0.0095 (12)	0.0089 (13)
C13B	0.0603 (16)	0.0697 (19)	0.0580 (16)	0.0013 (13)	0.0104 (13)	0.0109 (14)
C14B	0.0608 (16)	0.0608 (17)	0.0681 (18)	−0.0019 (13)	0.0126 (13)	0.0143 (14)
C15B	0.0634 (17)	0.0627 (18)	0.0690 (19)	0.0015 (13)	0.0094 (14)	0.0050 (14)
C16B	0.085 (2)	0.070 (2)	0.0606 (17)	0.0006 (16)	0.0186 (15)	0.0078 (15)
C17B	0.0721 (19)	0.0623 (18)	0.0706 (19)	0.0015 (14)	0.0195 (15)	0.0147 (15)
C18B	0.101 (3)	0.078 (2)	0.084 (2)	−0.0184 (19)	0.006 (2)	−0.0005 (18)
C19B	0.109 (3)	0.142 (4)	0.118 (4)	−0.041 (3)	0.002 (3)	−0.017 (3)
C20B	0.076 (2)	0.069 (2)	0.079 (2)	−0.0070 (16)	0.0113 (16)	0.0002 (16)
C21B	0.086 (2)	0.094 (3)	0.097 (3)	0.009 (2)	0.003 (2)	0.012 (2)
C22B	0.0940 (16)	0.0742 (14)	0.0833 (15)	−0.0112 (12)	0.0177 (12)	−0.0050 (11)
C23B	0.077 (2)	0.127 (3)	0.138 (4)	0.009 (2)	0.030 (2)	−0.006 (3)
C24B	0.111 (3)	0.083 (2)	0.074 (2)	−0.008 (2)	0.008 (2)	−0.0031 (18)
C25B	0.120 (4)	0.199 (6)	0.106 (3)	0.020 (4)	0.031 (3)	−0.002 (4)

### Geometric parameters (Å, º)

**Table:**

O1A—C9A	1.201 (4)	O1B—C9B	1.275 (3)
N1A—C3A	1.355 (4)	N1B—C3B	1.379 (4)
N1A—C20A	1.409 (5)	N1B—C18B	1.500 (4)
N1A—C18A	1.570 (5)	N1B—C20B	1.502 (4)
N2A—C15A	1.370 (5)	N2B—C15B	1.380 (4)
N2A—C24A	1.419 (5)	N2B—C22B	1.509 (4)
N2A—C22A	1.455 (5)	N2B—C24B	1.515 (4)
C1A—C2A	1.359 (5)	C1B—C2B	1.371 (4)
C1A—C6A	1.379 (4)	C1B—C6B	1.404 (4)
C1A—H1AA	0.9300	C1B—H1BA	0.9300
C2A—C3A	1.351 (4)	C2B—C3B	1.440 (4)
C2A—H2AA	0.9300	C2B—H2BA	0.9300
C3A—C4A	1.401 (4)	C3B—C4B	1.423 (4)
C4A—C5A	1.355 (4)	C4B—C5B	1.367 (4)
C4A—H4AA	0.9300	C4B—H4BA	0.9300
C5A—C6A	1.351 (4)	C5B—C6B	1.444 (4)
C5A—H5AA	0.9300	C5B—H5BA	0.9300
C6A—C7A	1.434 (4)	C6B—C7B	1.451 (4)
C7A—C8A	1.331 (4)	C7B—C8B	1.332 (4)
C7A—H7AA	0.9300	C7B—H7BA	0.9300
C8A—C9A	1.451 (4)	C8B—C9B	1.475 (4)
C8A—H8AA	0.9300	C8B—H8BA	0.9300
C9A—C10A	1.456 (5)	C9B—C10B	1.478 (4)
C10A—C11A	1.322 (4)	C10B—C11B	1.336 (4)
C10A—H10A	0.9300	C10B—H10B	0.9300
C11A—C12A	1.440 (5)	C11B—C12B	1.458 (4)
C11A—H11A	0.9300	C11B—H11B	0.9300
C12A—C17A	1.361 (4)	C12B—C13B	1.390 (4)
C12A—C13A	1.378 (4)	C12B—C17B	1.453 (4)
C13A—C14A	1.374 (5)	C13B—C14B	1.374 (4)
C13A—H13A	0.9300	C13B—H13B	0.9300
C14A—C15A	1.362 (5)	C14B—C15B	1.457 (4)
C14A—H14A	0.9300	C14B—H14B	0.9300
C15A—C16A	1.396 (5)	C15B—C16B	1.413 (4)
C16A—C17A	1.362 (5)	C16B—C17B	1.374 (4)
C16A—H16A	0.9300	C16B—H16B	0.9300
C17A—H17A	0.9300	C17B—H17B	0.9300
C18A—C19A	1.385 (7)	C18B—C19B	1.423 (6)
C18A—H18A	0.9700	C18B—H18C	0.9700
C18A—H18B	0.9700	C18B—H18D	0.9700
C19A—H19A	0.9600	C19B—H19D	0.9600
C19A—H19B	0.9600	C19B—H19E	0.9600
C19A—H19C	0.9600	C19B—H19F	0.9600
C20A—C21A	1.547 (7)	C20B—C21B	1.453 (5)
C20A—H20A	0.9700	C20B—H20C	0.9700
C20A—H20B	0.9700	C20B—H20D	0.9700
C21A—H21A	0.9600	C21B—H21D	0.9600
C21A—H21B	0.9600	C21B—H21E	0.9600
C21A—H21C	0.9600	C21B—H21F	0.9600
C22A—C23A	1.447 (7)	C22B—C23B	1.412 (5)
C22A—H22A	0.9700	C22B—H22C	0.9700
C22A—H22B	0.9700	C22B—H22D	0.9700
C23A—H23A	0.9600	C23B—H23D	0.9600
C23A—H23B	0.9600	C23B—H23E	0.9600
C23A—H23C	0.9600	C23B—H23F	0.9600
C24A—C25A	1.528 (7)	C24B—C25B	1.440 (6)
C24A—H24A	0.9700	C24B—H24C	0.9700
C24A—H24B	0.9700	C24B—H24D	0.9700
C25A—H25A	0.9600	C25B—H25D	0.9600
C25A—H25B	0.9600	C25B—H25E	0.9600
C25A—H25C	0.9600	C25B—H25F	0.9600
			
C3A—N1A—C20A	121.3 (3)	C3B—N1B—C18B	119.0 (3)
C3A—N1A—C18A	121.9 (3)	C3B—N1B—C20B	121.9 (2)
C20A—N1A—C18A	113.8 (3)	C18B—N1B—C20B	117.6 (3)
C15A—N2A—C24A	121.2 (3)	C15B—N2B—C22B	119.7 (3)
C15A—N2A—C22A	123.7 (4)	C15B—N2B—C24B	123.6 (3)
C24A—N2A—C22A	114.0 (4)	C22B—N2B—C24B	116.5 (3)
C2A—C1A—C6A	125.4 (3)	C2B—C1B—C6B	121.1 (3)
C2A—C1A—H1AA	117.3	C2B—C1B—H1BA	119.4
C6A—C1A—H1AA	117.3	C6B—C1B—H1BA	119.4
C3A—C2A—C1A	120.4 (3)	C1B—C2B—C3B	121.5 (3)
C3A—C2A—H2AA	119.8	C1B—C2B—H2BA	119.3
C1A—C2A—H2AA	119.8	C3B—C2B—H2BA	119.3
C2A—C3A—N1A	121.7 (3)	N1B—C3B—C4B	119.8 (3)
C2A—C3A—C4A	114.8 (3)	N1B—C3B—C2B	122.3 (3)
N1A—C3A—C4A	123.5 (3)	C4B—C3B—C2B	117.9 (3)
C5A—C4A—C3A	123.4 (3)	C5B—C4B—C3B	119.7 (3)
C5A—C4A—H4AA	118.3	C5B—C4B—H4BA	120.1
C3A—C4A—H4AA	118.3	C3B—C4B—H4BA	120.1
C6A—C5A—C4A	122.0 (3)	C4B—C5B—C6B	122.5 (3)
C6A—C5A—H5AA	119.0	C4B—C5B—H5BA	118.8
C4A—C5A—H5AA	119.0	C6B—C5B—H5BA	118.8
C5A—C6A—C1A	113.9 (3)	C1B—C6B—C5B	117.3 (3)
C5A—C6A—C7A	124.1 (3)	C1B—C6B—C7B	118.7 (3)
C1A—C6A—C7A	121.9 (3)	C5B—C6B—C7B	124.0 (3)
C8A—C7A—C6A	133.1 (3)	C8B—C7B—C6B	126.3 (3)
C8A—C7A—H7AA	113.4	C8B—C7B—H7BA	116.9
C6A—C7A—H7AA	113.4	C6B—C7B—H7BA	116.9
C7A—C8A—C9A	123.0 (3)	C7B—C8B—C9B	120.5 (3)
C7A—C8A—H8AA	118.5	C7B—C8B—H8BA	119.7
C9A—C8A—H8AA	118.5	C9B—C8B—H8BA	119.7
O1A—C9A—C8A	118.9 (3)	O1B—C9B—C8B	122.8 (3)
O1A—C9A—C10A	120.5 (3)	O1B—C9B—C10B	123.0 (3)
C8A—C9A—C10A	120.6 (3)	C8B—C9B—C10B	114.2 (3)
C11A—C10A—C9A	124.5 (3)	C11B—C10B—C9B	120.5 (3)
C11A—C10A—H10A	117.7	C11B—C10B—H10B	119.7
C9A—C10A—H10A	117.7	C9B—C10B—H10B	119.7
C10A—C11A—C12A	131.8 (3)	C10B—C11B—C12B	127.5 (3)
C10A—C11A—H11A	114.1	C10B—C11B—H11B	116.2
C12A—C11A—H11A	114.1	C12B—C11B—H11B	116.2
C17A—C12A—C13A	113.0 (3)	C13B—C12B—C17B	116.4 (3)
C17A—C12A—C11A	123.9 (3)	C13B—C12B—C11B	119.1 (3)
C13A—C12A—C11A	123.1 (3)	C17B—C12B—C11B	124.5 (3)
C14A—C13A—C12A	124.8 (3)	C14B—C13B—C12B	121.5 (3)
C14A—C13A—H13A	117.6	C14B—C13B—H13B	119.2
C12A—C13A—H13A	117.6	C12B—C13B—H13B	119.2
C15A—C14A—C13A	121.9 (3)	C13B—C14B—C15B	121.9 (3)
C15A—C14A—H14A	119.1	C13B—C14B—H14B	119.1
C13A—C14A—H14A	119.1	C15B—C14B—H14B	119.1
C14A—C15A—N2A	123.1 (3)	N2B—C15B—C16B	119.7 (3)
C14A—C15A—C16A	113.4 (3)	N2B—C15B—C14B	123.0 (3)
N2A—C15A—C16A	123.5 (3)	C16B—C15B—C14B	117.2 (3)
C17A—C16A—C15A	123.8 (3)	C17B—C16B—C15B	119.5 (3)
C17A—C16A—H16A	118.1	C17B—C16B—H16B	120.3
C15A—C16A—H16A	118.1	C15B—C16B—H16B	120.3
C12A—C17A—C16A	123.1 (3)	C16B—C17B—C12B	123.5 (3)
C12A—C17A—H17A	118.5	C16B—C17B—H17B	118.3
C16A—C17A—H17A	118.5	C12B—C17B—H17B	118.3
C19A—C18A—N1A	101.9 (5)	C19B—C18B—N1B	112.0 (4)
C19A—C18A—H18A	111.4	C19B—C18B—H18C	109.2
N1A—C18A—H18A	111.4	N1B—C18B—H18C	109.2
C19A—C18A—H18B	111.4	C19B—C18B—H18D	109.2
N1A—C18A—H18B	111.4	N1B—C18B—H18D	109.2
H18A—C18A—H18B	109.3	H18C—C18B—H18D	107.9
C18A—C19A—H19A	109.5	C18B—C19B—H19D	109.5
C18A—C19A—H19B	109.5	C18B—C19B—H19E	109.5
H19A—C19A—H19B	109.5	H19D—C19B—H19E	109.5
C18A—C19A—H19C	109.5	C18B—C19B—H19F	109.5
H19A—C19A—H19C	109.5	H19D—C19B—H19F	109.5
H19B—C19A—H19C	109.5	H19E—C19B—H19F	109.5
N1A—C20A—C21A	112.3 (4)	C21B—C20B—N1B	112.6 (3)
N1A—C20A—H20A	109.1	C21B—C20B—H20C	109.1
C21A—C20A—H20A	109.1	N1B—C20B—H20C	109.1
N1A—C20A—H20B	109.1	C21B—C20B—H20D	109.1
C21A—C20A—H20B	109.1	N1B—C20B—H20D	109.1
H20A—C20A—H20B	107.9	H20C—C20B—H20D	107.8
C20A—C21A—H21A	109.5	C20B—C21B—H21D	109.5
C20A—C21A—H21B	109.5	C20B—C21B—H21E	109.5
H21A—C21A—H21B	109.5	H21D—C21B—H21E	109.5
C20A—C21A—H21C	109.5	C20B—C21B—H21F	109.5
H21A—C21A—H21C	109.5	H21D—C21B—H21F	109.5
H21B—C21A—H21C	109.5	H21E—C21B—H21F	109.5
C23A—C22A—N2A	113.0 (5)	C23B—C22B—N2B	109.9 (3)
C23A—C22A—H22A	109.0	C23B—C22B—H22C	109.7
N2A—C22A—H22A	109.0	N2B—C22B—H22C	109.7
C23A—C22A—H22B	109.0	C23B—C22B—H22D	109.7
N2A—C22A—H22B	109.0	N2B—C22B—H22D	109.7
H22A—C22A—H22B	107.8	H22C—C22B—H22D	108.2
C22A—C23A—H23A	109.5	C22B—C23B—H23D	109.5
C22A—C23A—H23B	109.5	C22B—C23B—H23E	109.5
H23A—C23A—H23B	109.5	H23D—C23B—H23E	109.5
C22A—C23A—H23C	109.5	C22B—C23B—H23F	109.5
H23A—C23A—H23C	109.5	H23D—C23B—H23F	109.5
H23B—C23A—H23C	109.5	H23E—C23B—H23F	109.5
N2A—C24A—C25A	111.4 (4)	C25B—C24B—N2B	114.5 (4)
N2A—C24A—H24A	109.3	C25B—C24B—H24C	108.6
C25A—C24A—H24A	109.3	N2B—C24B—H24C	108.6
N2A—C24A—H24B	109.3	C25B—C24B—H24D	108.6
C25A—C24A—H24B	109.3	N2B—C24B—H24D	108.6
H24A—C24A—H24B	108.0	H24C—C24B—H24D	107.6
C24A—C25A—H25A	109.5	C24B—C25B—H25D	109.5
C24A—C25A—H25B	109.5	C24B—C25B—H25E	109.5
H25A—C25A—H25B	109.5	H25D—C25B—H25E	109.5
C24A—C25A—H25C	109.5	C24B—C25B—H25F	109.5
H25A—C25A—H25C	109.5	H25D—C25B—H25F	109.5
H25B—C25A—H25C	109.5	H25E—C25B—H25F	109.5
			
C6A—C1A—C2A—C3A	1.4 (5)	C6B—C1B—C2B—C3B	0.4 (5)
C1A—C2A—C3A—N1A	177.4 (3)	C18B—N1B—C3B—C4B	170.2 (3)
C1A—C2A—C3A—C4A	−1.4 (5)	C20B—N1B—C3B—C4B	4.4 (4)
C20A—N1A—C3A—C2A	−172.7 (4)	C18B—N1B—C3B—C2B	−10.3 (4)
C18A—N1A—C3A—C2A	−13.4 (6)	C20B—N1B—C3B—C2B	−176.1 (3)
C20A—N1A—C3A—C4A	6.0 (6)	C1B—C2B—C3B—N1B	179.5 (3)
C18A—N1A—C3A—C4A	165.3 (4)	C1B—C2B—C3B—C4B	−1.0 (4)
C2A—C3A—C4A—C5A	−0.1 (5)	N1B—C3B—C4B—C5B	179.8 (3)
N1A—C3A—C4A—C5A	−178.9 (3)	C2B—C3B—C4B—C5B	0.3 (4)
C3A—C4A—C5A—C6A	2.0 (5)	C3B—C4B—C5B—C6B	0.9 (4)
C4A—C5A—C6A—C1A	−2.0 (4)	C2B—C1B—C6B—C5B	0.7 (4)
C4A—C5A—C6A—C7A	176.5 (3)	C2B—C1B—C6B—C7B	−179.4 (3)
C2A—C1A—C6A—C5A	0.4 (5)	C4B—C5B—C6B—C1B	−1.4 (4)
C2A—C1A—C6A—C7A	−178.2 (3)	C4B—C5B—C6B—C7B	178.7 (3)
C5A—C6A—C7A—C8A	−2.1 (5)	C1B—C6B—C7B—C8B	−178.7 (3)
C1A—C6A—C7A—C8A	176.2 (3)	C5B—C6B—C7B—C8B	1.1 (5)
C6A—C7A—C8A—C9A	−179.9 (3)	C6B—C7B—C8B—C9B	178.9 (3)
C7A—C8A—C9A—O1A	−4.7 (5)	C7B—C8B—C9B—O1B	8.3 (5)
C7A—C8A—C9A—C10A	174.3 (3)	C7B—C8B—C9B—C10B	−171.6 (3)
O1A—C9A—C10A—C11A	4.6 (5)	O1B—C9B—C10B—C11B	1.2 (5)
C8A—C9A—C10A—C11A	−174.4 (3)	C8B—C9B—C10B—C11B	−178.8 (3)
C9A—C10A—C11A—C12A	176.3 (3)	C9B—C10B—C11B—C12B	−176.1 (3)
C10A—C11A—C12A—C17A	−0.9 (6)	C10B—C11B—C12B—C13B	179.6 (3)
C10A—C11A—C12A—C13A	−179.8 (3)	C10B—C11B—C12B—C17B	0.0 (5)
C17A—C12A—C13A—C14A	0.8 (5)	C17B—C12B—C13B—C14B	0.7 (4)
C11A—C12A—C13A—C14A	179.8 (3)	C11B—C12B—C13B—C14B	−179.0 (3)
C12A—C13A—C14A—C15A	1.1 (6)	C12B—C13B—C14B—C15B	1.0 (4)
C13A—C14A—C15A—N2A	177.3 (4)	C22B—N2B—C15B—C16B	171.2 (3)
C13A—C14A—C15A—C16A	−2.5 (5)	C24B—N2B—C15B—C16B	−13.0 (5)
C24A—N2A—C15A—C14A	−170.5 (4)	C22B—N2B—C15B—C14B	−5.9 (5)
C22A—N2A—C15A—C14A	−3.1 (7)	C24B—N2B—C15B—C14B	169.9 (3)
C24A—N2A—C15A—C16A	9.2 (7)	C13B—C14B—C15B—N2B	175.0 (3)
C22A—N2A—C15A—C16A	176.6 (5)	C13B—C14B—C15B—C16B	−2.2 (4)
C14A—C15A—C16A—C17A	2.3 (6)	N2B—C15B—C16B—C17B	−175.6 (3)
N2A—C15A—C16A—C17A	−177.5 (4)	C14B—C15B—C16B—C17B	1.7 (4)
C13A—C12A—C17A—C16A	−1.0 (5)	C15B—C16B—C17B—C12B	−0.1 (5)
C11A—C12A—C17A—C16A	179.9 (3)	C13B—C12B—C17B—C16B	−1.2 (4)
C15A—C16A—C17A—C12A	−0.5 (6)	C11B—C12B—C17B—C16B	178.4 (3)
C3A—N1A—C18A—C19A	104.1 (5)	C3B—N1B—C18B—C19B	96.1 (4)
C20A—N1A—C18A—C19A	−95.1 (5)	C20B—N1B—C18B—C19B	−97.5 (4)
C3A—N1A—C20A—C21A	79.5 (5)	C3B—N1B—C20B—C21B	79.9 (4)
C18A—N1A—C20A—C21A	−81.4 (5)	C18B—N1B—C20B—C21B	−86.1 (4)
C15A—N2A—C22A—C23A	106.8 (6)	C15B—N2B—C22B—C23B	90.3 (4)
C24A—N2A—C22A—C23A	−85.0 (6)	C24B—N2B—C22B—C23B	−85.8 (4)
C15A—N2A—C24A—C25A	75.1 (6)	C15B—N2B—C24B—C25B	97.6 (5)
C22A—N2A—C24A—C25A	−93.4 (5)	C22B—N2B—C24B—C25B	−86.5 (5)

### Hydrogen-bond geometry (Å, º)

*Cg*3 and *Cg*4 are the centroids of the C1B–C6B and C12B–C17B rings, respectively.

**Table:**

*D*—H···*A*	*D*—H	H···*A*	*D*···*A*	*D*—H···*A*
C8*B*—H8*BA*···O1*B*^i^	0.93	2.59	3.479 (4)	160
C16*A*—H16*A*···*Cg*3^ii^	0.93	2.91	3.758 (4)	152
C21*A*—H21*A*···*Cg*4^iii^	0.96	2.79	3.541 (5)	136

Symmetry codes: (i) *x*, −*y*+1/2, *z*−1/2; (ii) −*x*+1, *y*+1/2, −*z*+5/2; (iii) *x*+1, *y*+1, *z*.

## References

[bb1] Allen, F. H., Kennard, O., Watson, D. G., Brammer, L., Orpen, A. G. & Taylor, R. (1987). *J. Chem. Soc. Perkin Trans. 2*, pp. S1–19.

[bb2] Barnabas, M. V., Liu, A., Trifunac, A. D., Krongauz, V. V. & Chang, C. T. (1992). *J. Phys. Chem.* **96**, 212–217.

[bb3] Bruker (2009). *APEX2*, *SAINT* and *SADABS* Bruker AXS Inc., Madison, Wisconsin, USA.

[bb4] Fun, H.-K., Ruanwas, P. & Chantrapromma, S. (2010). *Acta Cryst.* E**66**, o307–o308. 10.1107/S1600536809055421 PMC2979664 21579739

[bb5] Harrison, W. T. A., Sarojini, B. K., Vijaya Raj, K. K., Yathirajan, H. S. & Narayana, B. (2006). *Acta Cryst.* E**62**, o1522–o1523.

[bb6] Macrae, C. F., Edgington, P. R., McCabe, P., Pidcock, E., Shields, G. P., Taylor, R., Towler, M. & van de Streek, J. (2006). *J. Appl. Cryst.* **39**, 453–457.

[bb7] Makarov, M. V., Leonova, E. S., Rybalkina, E. Yu., Khrustalev, V. N., Shepel, N. E., Roschenthaler, G.-V., Timofeeva, T. V. & Odinets, I. L. (2012). *Arch. Pharm. Chem. Life Sci.* **345**, 349–359. 10.1002/ardp.201100352 22213431

[bb8] Ruanwas, P., Chantrapromma, S. & Fun, H.-K. (2011). *Acta Cryst.* E**67**, o33–o34. 10.1107/S1600536810049299 PMC3050352 21522745

[bb9] Sheldrick, G. M. (2008). *Acta Cryst.* A**64**, 112–122. 10.1107/S0108767307043930 18156677

[bb10] Shibata, H., Yamakoshi, H., Sato, A., Ohori, H., Kakudo, Y., Kudo, C., Takahashi, Y., Watanabe, M., Takano, H., Ishioka, C., Noda, T. & Iwabuchi, Y. (2009). *Cancer Sci.* **100**, 956–960. 10.1111/j.1349-7006.2009.01127.x PMC11158762 19445025

[bb11] Spek, A. L. (2009). *Acta Cryst.* D**65**, 148–155. 10.1107/S090744490804362X PMC2631630 19171970

[bb12] Wanare, G., Aher, R., Kawathekar, N., Ranjan, R., Kaushik, N. K. & Sahal, D. (2010). *Bioorg. Med. Chem. Lett.* **20**, 4675–4678. 10.1016/j.bmcl.2010.05.069 20576433

[bb13] Weber, W. M., Hunsaker, L. A., Abcouwer, S. F., Decka, L. M. & Vander, D. L. (2005). *Bioorg. Med. Chem.* **13**, 3811–3820. 10.1016/j.bmc.2005.03.035 15863007

[bb14] Westrip, S. P. (2010). *J. Appl. Cryst.* **43**, 920–925.

[bb15] Zhao, C., Yang, J., Wang, Y., Liang, D., Yang, X., Li, X., Wu, J., Wu, X., Yang, S., Li, X. & Liang, G. (2010). *Bioorg. Med. Chem.* **18**, 2388–2393. 10.1016/j.bmc.2010.03.001 20338767

